# Mesoporous Silica Nanoparticles Modified inside and out for ON:OFF pH-Modulated Cargo Release

**DOI:** 10.3390/pharmaceutics13050716

**Published:** 2021-05-13

**Authors:** José L. M. Gonçalves, Ana Beatriz C. Lopes, Carlos Baleizão, José Paulo S. Farinha

**Affiliations:** Centro de Química Estrutural and Department of Chemical Engineering, Instituto Superior Técnico, Universidade de Lisboa, 1049-001 Lisboa, Portugal; joseluis@tecnico.ulisboa.pt (J.L.M.G.); ana.beatriz.lopes6@gmail.com (A.B.C.L.)

**Keywords:** core-shell hybrid mesoporous silica nanoparticles, pH-responsive polymers, modulated cargo release, pore functionalization, RAFT

## Abstract

Highly efficient pH-modulated cargo release was achieved with a new hybrid nanocarrier composed of a mesoporous silica core with functionalized pores and a grafted pH-responsive crosslinked polymer shell of 2-(diisopropylamino)ethyl methacrylate (pKa ≈ 6.5). The retention/release performance of the system was optimized by a novel approach using selective functionalization of the silica pores to tune the carrier-cargo interaction and by tunning the amount of grafted polymer. The system features excellent retention of cationic cargo at low pH and a burst release at higher pH. This results from the expanded-collapsed conformation transition of the pH-responsive polymer shell and the simultaneous change in the interaction between the cargo and the polymer shell and the modified pore walls. At low pH, the electrostatic interaction of the cationic cargo with the protonated amine groups of the extended polymer shell retains the cargo, resulting in very low leakage (OFF state). At high pH, the electrostatic interaction with the cargo is lost (due to deprotonation of the polymer amine groups), and the polymer shell collapses, squeezing out the cargo in a burst release (ON state). Pore functionalization in combination with the stimuli-responsive polymer shell is a very promising strategy to design high-performance ON:OFF smart hybrid nanocarriers for stimuli-actuated cargo release, with great potential for application in the controlled release of drugs and other biologically active agents.

## 1. Introduction

Response to stimuli is a prevalent phenomenon in life, not only as a basic functioning process in all living systems but also affecting the behavior of higher organisms [1]. Artificial functional intelligent materials are designed to recognize a specific environmental stimulus and respond to this stimulus with a predetermined and useful output, returning to the original state in the absence of the stimulus. Many types of stimuli have been reported, both chemical (pH, redox, solvent, and ionic strength), physical (temperature, light, magnetic, mechanical, and electric), and biological (enzymatic, receptor-ligand recognition, etc.). Different types of response have also been explored, from changes in conformation or adherence to providing chemical and biochemical cues to cells [2]. Furthermore, these systems are capable of actuating without a dedicated energy supply [3].

One very promising application of stimuli-responsive materials is the controlled delivery of substances at the nanoscale. The response of these materials to a given stimulus can provide the mechanical and chemical changes that modulate the diffusion of the cargo from the nanodevice. Such a control allows the development of systems that release their payload at a targeted location, in the desired amount and in a timely manner, thus increasing the efficiency of such cargo by protecting it while in transit and using only the necessary amount [4,5].

Recent advances in the design of intelligent materials, in particular smart polymers, have led to systems that can respond to a dynamic environment, with the fluctuation of stimuli over time inducing a modulated response from the devices [6,7,8,9]. In the case of smart polymer materials, this is achieved by using the changes in polymer chain conformation and interactions induced by a trigger, such as a temperature [10,11,12], pH [13], light [14], proteins [15], ionic strength [16], etc., or combinations thereof [17].

The response to a change in pH is particularly interesting. Smart polymers responding to pH can be designed to change conformation and charge at a predetermined pH value, which can be chosen by selecting the appropriate monomer units [18,19]. This allows the development of systems programmed for pH-activated cargo delivery or pick-up at locations with specific acidity conditions. Such systems have very promising applications, from regulating fertilizer release to increase agricultural productivity minimizing damage to the soil and the ecosystems [20], to environmental remediation [21,22] and metal harvesting from water [23], corrosion control [20,24,25,26,27,28], drug delivery [29], etc.

However, the more promising application of pH-responsive systems is probably in the precise control of drug delivery, where these systems have been used to optimize drug pharmacokinetics, avoid accumulation in non-target tissues, and minimize side effects [4]. To improve the accumulation of the carriers at the desired location, these can be functionalized with specific groups that interact with receptors overexpressed at the desired cells or tissues [30,31].

pH-triggered drug delivery is especially useful for self-regulated drug delivery since normal and non-healthy tissues usually feature detectable differences in pH [32]. Cancer tissues generally have a more acidic extracellular environment than the corresponding healthy ones [33], leading to the development of numerous low-pH releasing systems. However, high-pH releasing strategies can also be of interest since recent results have shown that intracellular pH is neutral or even mildly alkaline compared to normal cells [33]. Release at higher pH can also be of interest to design nanocarriers that retain their cargo during a gastric passage and release it in the alkaline intestinal tract. The positively charged polymer shell can facilitate loading and trapping of anionic drugs (such as sulfasalazine, an anti-inflammatory prodrug for bowel disease), with deprotonation leading to electrostatic repulsion-triggered sustained release [34,35].

Delivery systems based on polymer alone generally have low cargo capacity and poor chemical and mechanical properties (degradable polymers) or poor biocompatibility. Therefore, to take full advantage of the potential of stimuli-responsive polymers, these have been grafted to the surface of more robust nanocarriers [6,7,8]. Periodically ordered mesoporous silica materials, developed at Mobil by using surfactant micelles to template the condensation of reactive silica precursors [36], have been downsized to create mesoporous silica nanoparticles (MSNs), which are used as nanoscale carriers, in particular in drug delivery applications [37,38,39]. MSNs offer numerous design advantages for the development of smart nanocarriers, including high mechanical stability, well-defined particle morphology, tunable particle diameter (from 15 nm to several hundred nanometers) [40], tunable pore size (2–8 nm in diameter) and pore geometry (ordered/worm-like), and good colloidal stability [40,41,42,43]. MSNs also offer different tracking options by incorporating in their structure fluorescent dyes or quantum dots, iron oxide nanocrystals or gadolinium ions, gold or silver nanoparticles, among others, so that the MSNs can be imaged by optical microscopy, magnetic resonance imaging, ultrasonic imaging, or other techniques [44]. MSNs have very high cargo loading capacity due to their internal surface area and available pore volume (>1 mL/g) and allow a large diversity of surface functionalization options.

One seldomly used advantage of MSNs is that they can be selectively and independently functionalized on both their inner (pore) and outer surfaces. While the functionalization of the outer surface has been extensively used to covalently attach stimuli-responsive polymers or (bio)molecules for controlled release, destination targeting, etc., the possibility of inner pore surface modification has not been fully explored. The latter can be used to control the interaction of the solvent and the cargo with the pore walls, opening new opportunities for cargo delivery control [45].

Here, we report novel smart hybrid nanocontainers composed of a fluorescent mesoporous silica nanoparticle (MSN) core with the pores modified with groups that increase the interaction with the cargo and a gel-like pH-responsive cross-linked polymer shell of poly(2-(diisopropylamino)ethyl methacrylate) (pDAEM). This polybase of a tertiary amine methacrylate has pKa ~ 6.5 [18], which means that the polymer shell is in an expanded conformation at acidic pH and collapses onto the MSN core in alkaline conditions. A fluorescent perylenediimide derivative (PDI) was incorporated in the silica structure for optical tracking of the nanoparticles, followed by modification of the pore surface, and finally, the polymer shell was grown (grafted) from the MSN surface by reverse addition-fragmentation transfer (RAFT) polymerization to allow good control of the gel thickness. This new strategy allows us to design a trackable system with very efficient cargo release (ON) and cargo retention (OFF) states.

The performance of the cargo loading and controlled release was tested using a model fluorescent molecule, sulforhodamine B (SRB), by continuously monitoring the fluorescence intensity of the released cargo as we modulate the pH of the media. By modifying the pore surfaces with a cationic group, the polymer shell thickness could be reduced, avoiding the cargo release barrier effect of the systems with higher polymer gel content, which slows cargo delivery during the releasing period. This approach yielded excellent ON:OFF performance with fast ON:OFF cycles. Our novel smart hybrid nanocarriers show very high potential for use in pH-actuated modulated cargo delivery applications.

## 2. Materials and Methods

### 2.1. Materials

*N*-cetyltrimethylammonium bromide (CTAB, 99% Sigma-Aldrich), absolute Ethanol (99.9% EtOH, Scharlau), hydrochloric acid (HCl, 37%, AnalaR NORMAPUR—VWR), sodium hydroxide (NaOH, pure EKA Pellets, Bohus), tetraethoxysilane (TEOS, 99%, Sigma-Aldrich), 3-aminopropyl triethoxysilane (APTES, 98%, Sigma-Aldrich), *N*-(3-Dimethylaminopropyl)-*N*′-ethylcarbodiimide (EDC, 98%, SigmaAldrich), *N*-trimethoxysilylpropyl- *N*, *N*, *N*-trimethylammonium chloride (CAT, 50% ethanol, Gelest), ethylene glycol dimethacrylate (EGDMA, 98%, Sigma-Aldrich), and 4-Cyano-4-(phenylcarbonothioylthio) pentanoic acid (CPADB, 97%, Sigma-Aldrich) were used as received. Azobisisobutyronitrile (AIBN, 99%, Sigma-Aldrich) was recrystallized from trifluoroacetic acid (>99%, Merck), and 1,4-dioxane (>99.9%, Sigma-Aldrich) and absolute ethanol (99.9% EtOH, Scharlau) were used as received. Disodium hydrogen phosphate (Na_2_HPO_4_, 99%, Riedel-de-Haen), sodium dihydrogen phosphate monohydrate (NaH_2_PO_4_, 98%, Panreac), and sodium hydroxide (NaOH, 98%, SigmaAldrich) were used to prepare the phosphate buffer solutions (PBS, 100 mM pH 5 and pH 9). Toluene, triethylamine, and dichloromethane were distilled over calcium hydride prior to use. Tetrahydrofuran (99% THF, Aldrich) was distilled over sodium prior to use. Sulforhodamine B (SRB, Molecular Probes) was used as a model cargo for the release studies in a polypropylene dialysis device with a cellulose membrane (Slide-A-Lyzer Mini Dialysis Devices, 10 K MWCO). The perylenediimide derivative (PDI) [33] and monomer 2–(diisopropylamino) ethyl methacrylate (DAEM) [42] were synthesized according to the literature. Deionized water from a Millipore system Milli-Q ≥ 18 MΩ cm (with a Millipak membrane filter 0.22 µm) was used in the preparation of solutions and in synthesis.

### 2.2. Methods

#### 2.2.1. Synthesis of Mesoporous Silica Nanoparticles (MSNs)

MSNs were synthesized following a method previously reported [40]. In a polypropylene flask, PDI (6.0 mg) and CTAB (0.500 g) were mixed in THF (5 mL). The mixture was sonicated for 15 min and left stirring at 40 °C until THF was evaporated. In a 500 mL polypropylene flask, deionized (DI) water (240 mL) and a NaOH solution (1.7 M, 1.75 mL) were added. The solid mixture CTAB/PDI was added to the flask at 32 °C. Afterwards, TEOS (2.5 mL) was added dropwise, and the solution was left stirring for 3 h. The particles were recovered by centrifugation, and the solid was washed with a mixture of ethanol and water. The solid obtained was dried at 50 °C overnight and later under vacuum.

#### 2.2.2. MSNs Amine Functionalization (MSN-NH_2_)

The MSNs containing the template (MSN-CTAB, 0.6 g) were dispersed in a solution of APTES (0.15 mL) in dry toluene (22 mL). The reaction mixture was kept at 130 °C under an argon atmosphere for 24 h. The nanoparticles were recovered by centrifugation and washed with ethanol. For the template removal, the nanoparticles were re-suspended and sonicated (15 min) in an acidic ethanol solution ([HCl] = 0.5 M, 25 mL per 500 mg of particles). The mixture was left under stirring at 50 °C for 24 h, and the nanoparticles were recovered by centrifugation and washed with a basic ethanol solution (NH_4_ 25%*v*/*v*) and ethanol. The solid (MSN-NH_2_) was dried at 50 °C for 24 h.

#### 2.2.3. Surface Modification with a RAFT Agent (MSN-CTA)

MSN-NH_2_ was dispersed in dry dichloromethane (23 mL) under an argon atmosphere and sonicated for 20 min. Then, EDC (1.2 eq. to APTES) and CPADB (1 eq. to APTES) were added to the mixture and left stirring at room temperature for 24 h. MSN-CTA was recovered by centrifugation and washed with ethanol. The product was dried at 50 °C for 24 h.

#### 2.2.4. Pore Functionalization (MSN-CAT-CTA)

MSN pore surface functionalization was performed using a cationic alkoxysilane, *N*-trimethoxysilylpropyl- *N*,*N*,*N*-trimethylammonium chloride (CAT). MSN-CTA (0.2 g) was dispersed in a solution of dry toluene (10 mL) and CAT (1.1 mL). The mixture was kept at 35 °C with an argon atmosphere for 72 h. MSN-CAT-CTA nanoparticles were recovered by centrifugation and washed with ethanol. The solid was dried at 50 °C for 24 h.

#### 2.2.5. Polymer Grafting to the MSN Surface (MSN-pDAEM)

The preparation of the MSN-pDAEM using the grafting-from method was carried out according to the following steps. In a Schlenk flask, MSN-CTA nanoparticles (40 mg) ([CTA] = 0.04 mmol·g^−1^) were added under argon atmosphere. In another Schlenk (B), DAEM monomer (1 eq.), EDGMA (0.04 eq.), TFA (1.5 eq.), an AIBN ethanolic solution (adjusted for the target [AIBN]/[CTA] ratio), and ethanol (adjusted for the target DPA concentration) were added under an argon atmosphere. Oxygen was removed from the mixture after five freeze-pump cycles. The mixture was transferred from (B) to (A) with a cannula and then place in an oil bath at 80 °C for 24 h. The core–shell nanoparticles (MSN-pDAEM) were recovered by centrifugation and washed with ethanol (13,600× *g*, three cycles of 10 min each). The particles were dried at 50 °C overnight and later under vacuum. The MSN-CAT-pDAEM nanocarriers were prepared using the same procedure but using MSN-CAT-CTA for the polymerization of the crosslinked polymer shell.

#### 2.2.6. Loading and Release of SRB

A physical entrapment method was used to load the cargo molecules in the MSN pore structure. SRB was used as a model cargo molecule to test the load/release capability of the hybrid MSN-pDAEM. An ethanolic solution of SRB (3.90 × 10^−3^ M, 500 µL) was added to dry MSN-pDAEM (1.5 mg), and the dispersion was stirred overnight at room temperature. The dispersion was centrifuged to remove the unloaded SRB, and the nanoparticles were redispersed twice in phosphate buffer (1 mL, pH 9) and centrifuged. After the last centrifugation, the supernatant was removed, and 200 µL of PBS (pH 5) was added to the loaded nanoparticles. The mixture was transferred to the dialysis device and inserted on top of the fluorescence cuvette (previously filled with 3.2 mL of PBS (pH 5)) immediately before the beginning of the experiment. SRB released from the nanoparticles was monitored through the SRB fluorescence intensity on the bottom compartment of the fluorescence cuvette, while modulating the pH between 5 and 9, with additions of NaOH (1 M, 21 µL) (3690 s, 10,560 s, and 26,210 s) for 8.3 h. The supernatants were recovered and used to determine the loading efficiency, by UV–Vis absorption: 0.0094 mmol·g^−1^ for MSN-pDAEM2, 0.011 mmol·g^−1^ for MSN-pDAEM5 and 0.0084 mmol·g^−1^ for MSNpDAEM15 [7].

### 2.3. Characterization Methods

#### 2.3.1. UV-Vis Spectroscopy

UV-660 UV–VIS Spectrophotometer (JASCO International, Tokyo, Japan), supplied with a double monochromator and a photomultiplier detector for higher resolution. pH measurement: bench pH/mV/°C meter pH 1000 L, pHenomenal^®^.

#### 2.3.2. Transmission Electronic Microscopy (TEM)

Hitachi transmission electron microscope (Hitachi High-technologies, Tokyo, Japan), model H-8100, with a LaB_6_ filament (Hitachi High-Technologies Europe GmbH, Krefeld, Germany) complemented with an accelerator voltage of 200 kV and a current of 20 µA. A camera KeenView (Soft Imaging System, Münster, Germany) is incorporated in this equipment, which through the iTEM software allows acquiring TEM images. MSN dispersed in ethanol were prepared and dried on a Formvar carbon-coated copper grid 200 mesh (Ted Pella, Redding, CA, USA).

#### 2.3.3. Adsorption–Desorption Isotherms

The N_2_ adsorption–desorption isotherms were obtained at 77 K for the degassed samples in a gas porosimeter with an accelerated surface area and porosimetry system (Micromeritics, model ASAP 2010, Norcross, GA, USA).

#### 2.3.4. Nanoparticle Tracking Analysis (NTA)

It was used to measure the hydrodynamic diameter of the hybrid nanocarriers, using an LM20 System equipped with a 405 nm diode laser (NanoSight, Salisbury, UK). Diluted samples (106 to 109 particles per mL) of the nanocarriers were injected into the sample chamber. Videos of the scattering particles undergoing Brownian motion were captured and analyzed with NanoSight NTA 2.3 Analytical Software. All samples were measured in the single shutter and gain mode at 20 °C. Fifty 10 s measurements of each sample were performed, with a newly injected sample for each run. The diameter distributions were calculated from the sizes of all particles analyzed.

#### 2.3.5. Zeta-Potential

The Zetasizer Nano ZS (Malvern, model ZEN3600) was used. Zeta potentials were calculated from electrophoretic mobility using the Smoluchowski relationship. Disposable folded capillary cells (DTS1070) were used for the measurement of zeta potentials. All measurements were performed in triplicate.

#### 2.3.6. Fluorescence Measurements

Horiba-JobinYvon (Kyoto, Japan) Fluorolog-3 spectrofluorometer using a fluorescent cell. Right angle geometry was used in all measurements (*λ*_exc_ = 566 nm, *λ*_em_ = 589 nm).

## 3. Results and Discussion

**Figure 1:**
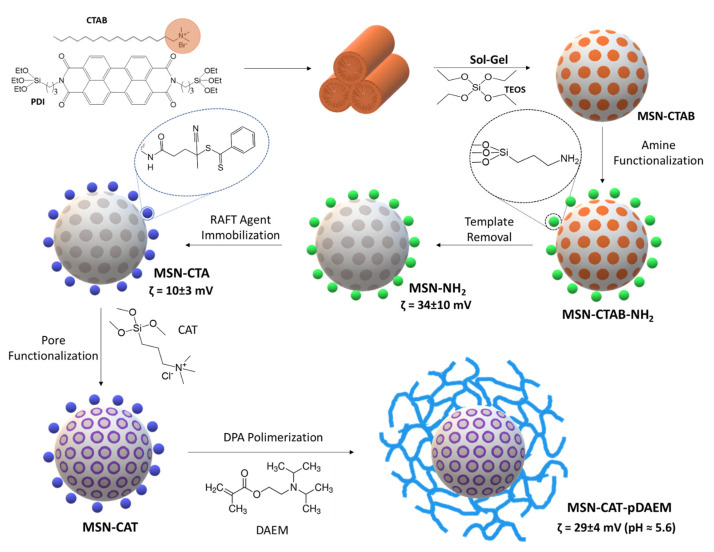
Preparation of hybrid fluorescent core-shell MSNs with functionalized pores and a pH-responsive polymer shell. The fluorescent dye (PDI) is anchored to the silica structure (MSN-CTAB), which is then surface-modified with amine groups (MSN-CTAB-NH_2_). After template removal (MSN-NH_2_), a RAFT CTA is immobilized in the surface by reaction with the amine groups. The free pores are then functionalized with a cationic group, and the CTA is used to polymerize the shell by RAFT. Zeta-potential values measured at pH = 5.6 reflect the change in surface charge upon surface modification.

Our strategy to develop the pH-actuated hybrid nanocarriers relies on the synthetic versatility of mesoporous silica nanoparticles (MSNs). Their external surface can be functionalized with a chain transfer agent (CTA) for reverse addition-fragmentation transfer (RAFT) controlled-radical polymerization of a pH-responsive gel from the MSN surface. After removal of the template agent, the pore surfaces at their interior can, in turn, be modified with groups that allow us to control the interaction with the cargo to be transported. The CTA was then used to grow a homogeneous polymer shell covalently bound to the MSN core by RAFT polymerization in order to maximize the efficiency of cargo release control (Figure 1).

### 3.1. Nanocarrier Assembly

**Figure 2:**
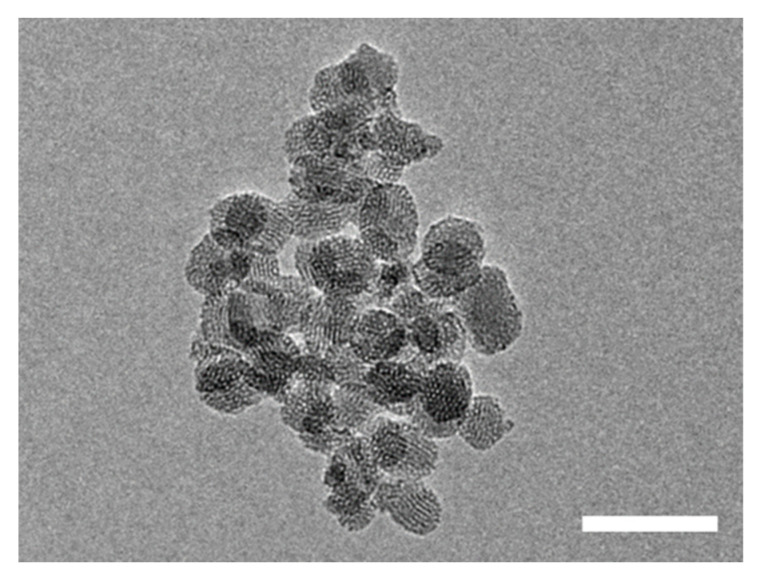
TEM image of the MSNs, with ordered pore structure and an average diameter of (45 ± 9) nm (obtained from the analysis of 50 particles in TEM images). Scale bar = 100 nm.

The particles were first prepared by a recent aqueous low-temperature sol-gel process that allows full control over their size [40,46]. The obtained nanoparticles have an aligned pore structure visible in the TEM images (Figure 2), with an average diameter of (45 ± 9) nm. The nanoparticles present a well-defined pore structure (Figure 2), with nitrogen sorption yielding a type IV isotherm (typical of this type of materials, Figure S1, Supporting Information). From BET analysis, we calculated a surface area of 530 m^2^ g^−1^, and using the Barrett–Joyner–Halenda (BJH) method, we obtained a pore volume of 0.35 cm^3^ g^−1^ with a pore diameter of 2.4 nm. MSNs produced by the same process were characterized by HR-TEM and high angular annular dark-field STEM, confirming their pore size and organization [42].

The external and internal surfaces of the silica nanoparticles can be independently modified by covalently attaching molecules containing alkoxysilane groups. Before template removal, the external surface was selectively functionalized (while the internal surface is “protected” by the template that occupies the pores). After template removal, the pores are, in turn, available for surface modification (or to be directly used for cargo loading).

**Figure 3:**
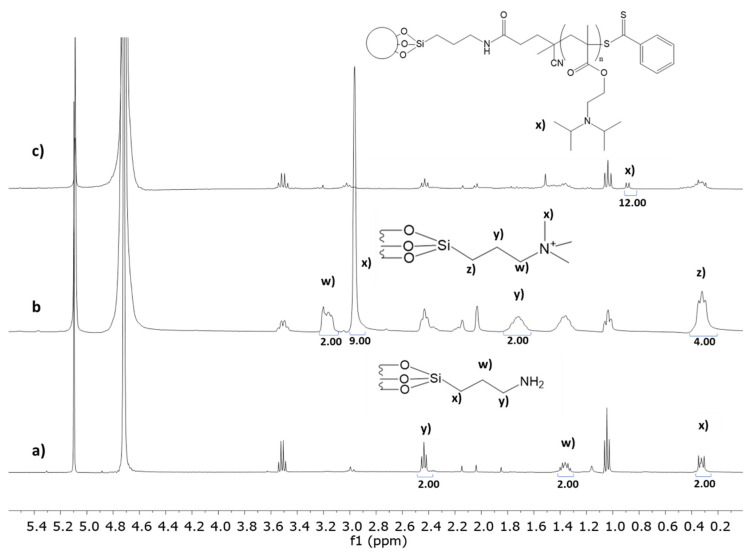
Solution ^1^H-NMR of (**a**) MSN-NH_2_, (**b**) MSN-CAT, and (**c**) of MSN-pDAEM (at pH = 13) in D_2_O. Trioxane (5.17 ppm, internal standard, C_3_H_6_O) was used as an internal reference. Ethanol peaks (from the washings) at 1.0 and 3.5 ppm, and D_2_O peak at 4.7 ppm.

To add a pH-responsive polymer shell to the MSN, we used a graft-from controlled RAFT polymerization approach (gRAFT-*from*) [47]. The MSNs were surface-modified with APTES before template removal to incorporate amine groups on their external surface, which are later used to immobilize a chain transfer agent (CTA) used to grow the polymer. The amine concentration at the MSN surface was 0.84 mmol·g^−1^ (determined by ^1^H NMR) [48], corresponding to a surface density of 1.7 amine groups per nm^2^ (Figure 3a). After removing the template with an acidified ethanolic solution, the CTA agent was immobilized by reaction with the amine groups in the presence of EDC. The amount of immobilized CTA was 0.08 mmol·g^−1^ (determined by UV-Vis spectroscopy) [31], corresponding to a surface density of 0.16 molecules per nm^2^ (Figure S2, Supporting Information). This low density minimizes termination reactions in the early stages of the polymerization and consequently allows the incorporation of higher polymer content at the particle surface [49]. The success of the previous steps was confirmed by zeta-potential measurements (Figure 1), with variations upon immobilization reactions indicating a clear change in the nanoparticles’ external surface charge.

After the modification of the external surface with the CTA, the pores were functionalized with a cationic molecule, *N*-trimethoxysilylpropyl-*N*,*N*,*N*-trimethylammonium chloride (CAT). The quantification by ^1^H NMR (Figure 3b) indicates a CAT concentration per gram of silica of 0.73 mmol·g^−1^.

The polymer shell was then grown from the surface of the nanoparticles by grafting from RAFT polymerization. If used to prepare a polymer brush (linear polymer chains), this process yields low polymer incorporation due to steric hindrance and termination reactions between the growing polymer chains [50,51]. One way to circumvent this problem is to use a cross-linking agent so that the growing polymer has lower mobility, and consequently, the termination rate is reduced, leading to a thicker gel-like polymer shell [47].

The polymerization was performed using different monomer concentrations and (CTA)/(Initiator) ratios to optimize the quantity of incorporated polymer. The (CTA)/(Initiator) ratio is related to the number of chains initiated at the particles’ surface (CTA) or in solution (Initiator). Therefore, higher (CTA)/(Initiator) ratios contribute to higher polymer incorporation in the nanoparticles, as shown by our previous results [47]. By using 10:1 (CTA)/(Initiator), we obtained 2.1 wt% and 5.1 wt% of polymer shell (polymer relative to silica weight) for reactions with 0.5 M of monomer (MSN-pDAEM2) and 2 M of monomer (MSN-pDAEM5), respectively. This corresponds to the double of the polymer incorporation achieved with a 5:1 (CTA)/(Initiator) ratio (Table S1, Supporting Information). The amount of polymer was determined by ^1^H-NMR after destroying the silica core at basic pH and using trioxane as an internal standard (Figure 3c) [48]. The results also show that higher monomer concentration increases the polymer incorporation so that in a polymerization reaction with 8 M monomer and a 10:1 (CTA)/(Initiator) ratio, we obtained 15 wt% of polymer shell (MSN-pDAEM15).

The hybrid nanoparticles are sensitive to TEM experimental conditions, with the polymeric shell being visible only for the MSN-pDAEM15 sample (Figure S3, Supporting Information), and with evident signs of polymer melting (the roughly estimated polymer shell thickness values are 15 nm, 7 nm, and 3 nm for MSN-pDAEM15, MSNpDAEM5, and MSN-pDAEM2, respectively). The zeta-potential of the hybrid nanoparticles, MSNpDAEM, is completely distinct from that of CTA-modified MSNs, MSN-CTA, confirming the formation of the polymer shell (Figure 1). The hydrodynamic diameter (*D*_H_) of the polymer-coated MSNs was measured by Nanoparticle Tracking Analysis (NTA) at pH values lower than the pKa of the monomer (~6.5), where the polymer is in an expanded conformation. The obtained values, *D*_H_ = 75 nm for MSNpDAEM2, *D*_H_ = 105 nm for MSN-pDAEM5 and *D*_H_ = 115 nm for MSN-pDAEM15 (Figure S4, Supporting Information), are larger than the core size obtained by TEM (45 nm, Figure 2) and increase with the polymer content as expected, but are probably affected by particle flocculation during the measurements.

### 3.2. Cargo Relase Modulation

To individually test the different controlling mechanisms for cargo release from our nanocarrier, we used sulforhodamine B (SRB) as model cargo. SRB has a high fluorescence quantum yield (40%) that is not affected by pH changes at least for pH 3–10 [52,53], and is water-soluble, allowing simple and reliable in-line quantification down to nanomolar concentrations [7]. We tested three different systems: (i) silica nanoparticles with the pores functionalized with CAT; (ii) core-shell MSN-pDAEM nanoparticles with three different polymer shell thickness but no pore modification; (iii) core-shell MSN-pDAEM nanoparticles with the pores functionalized with CAT.

The cargo was loaded into previously dried nanocarriers, which were dispersed in a concentrated ethanolic solution of SRB. During this process, the polymer of the hybrid particles is expanded, and the SRB diffuses into the pores. The nanoparticles were then washed with PBS at pH 9 to remove the SRB molecules adsorbed at the nanoparticles’ outer surface [7]. At this pH, the polymer shell in the hybrid nanoparticles is collapsed (pH higher than the pKa of the monomer), thus minimizing loss of the cargo in these systems. The amount of SRB loaded was determined from the supernatants by UV-Vis absorption, yielding ca. 0.01 mmol·g^−1^.

#### 3.2.1. Functionalized Pore Strategy

**Figure 4:**
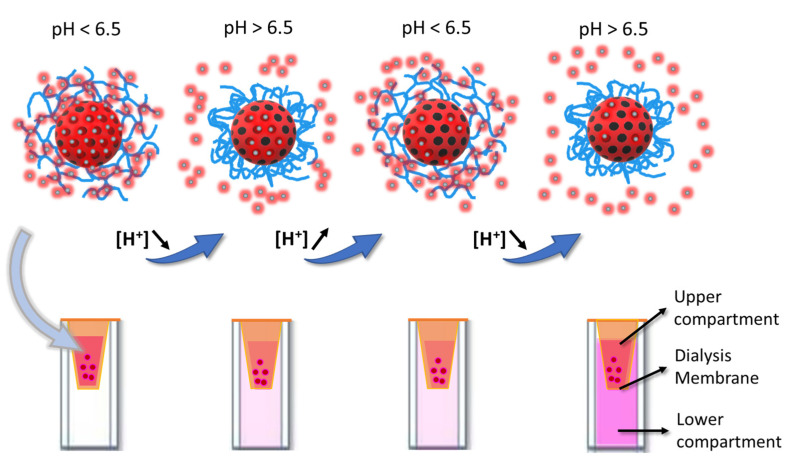
Schematic representation of SRB (black/red dots) release from SRB-loaded nanocarriers. The polymer is expanded at low pH values and collapses by increasing the pH. When the polymer is expanded (low pH), SRB diffuses from the pores but is trapped in the polymer network due to the electrostatic interaction with the protonated amine groups of the polymer shell. By increasing the pH, the cargo is released because the polymer collapses and the electrostatic interaction with the cargo is lost due to deprotonation of the polymer amine groups. By modulating the pH value, the process can be repeated. The system behaves like a nanopump, squeezing out the cargo with a sponge-like mechanism in response to the pH modulation.

MSNs without a polymer shell, with and without functionalization of the pore walls with CAT, were loaded with SRB, and the release of the cargo was followed in real-time, using a fluorescence cuvette with a top chamber containing the particle dispersion, separated from the bottom measuring compartment by a dialysis membrane (Figure 4). Unlike the nanoparticles, the released SRB can diffuse across the membrane to the bottom compartment, where the fluorescence intensity of the SRB is monitored. With this setup, we avoid the interference of light scattering from the nanoparticles and separate the fluorescence emission of the released SRB from that of SRB in the MSN pores.

**Figure 5:**
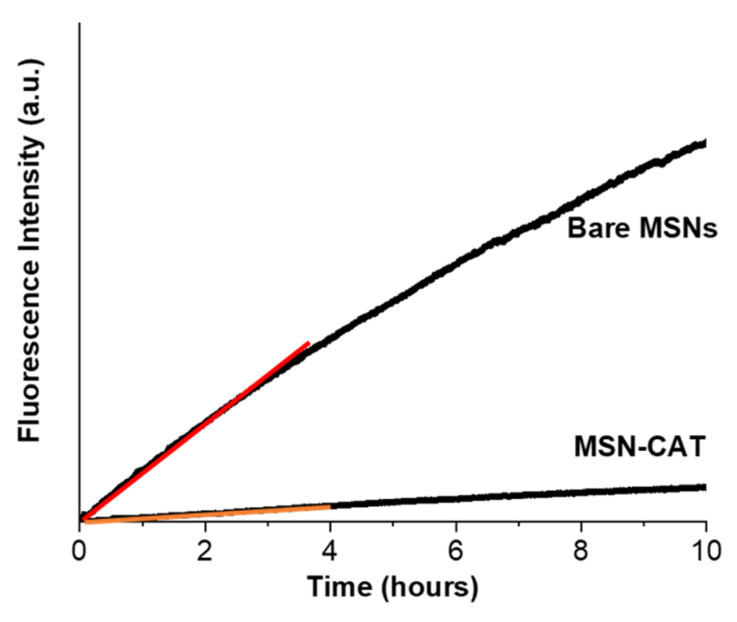
Fluorescence intensity of SRB released from SRB-loaded bare MSNs and MSN-CAT, which diffuses across the dialysis membrane (measured in the bottom compartment of the cuvette, in PBS). The system is followed continuously for 10 h (λ_exc_ = 566 nm, λ_em_ = 589 nm).

The experiment was initiated with the SRB-loaded nanoparticles in a PBS solution (pH = 7.5) at the top compartment of the fluorescence cuvette, and the fluorescence intensity was monitored for 10 h. The pH of the PBS solution (pH = 7.5) was maintained throughout the experiment. The results for both functionalized (MSN-CAT) and non-functionalized (bare MSNs) nanoparticles (Figure 5) show that, even though the release profiles are similar, the amount of SRB released by MSN-CAT is 10 times lower than from the bare MSNs. This confirms that tuning the interaction between the pore walls and the cargo by appropriate pore wall functionalization is an efficient strategy to control cargo retention in the MSN nanocarriers.

#### 3.2.2. Core-Shell System

**Figure 6:**
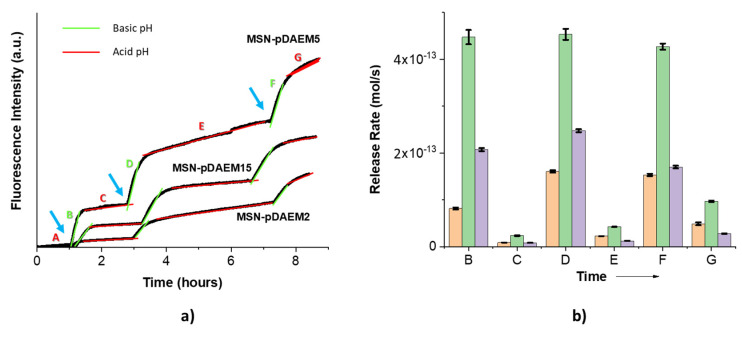
(**a**) Fluorescence intensity of SRB released from SRB-loaded MSN-pDAEM2, MSN-pDAEM5, and MSN-pDAEM15 in PBS (solid black lines). The released SRB diffuses across the dialysis membrane and is measured in the bottom compartment of the cuvette. The system is followed continuously for 8.3 h (λ*_exc_* = 566 nm, λ*_em_* = 589 nm). The blue arrows indicate additions of base solution in the top compartment of the cuvette, green lines (zones B, D, and F), and red lines (zones C, E, and G) indicate basic and acid pH periods in the top compartment, respectively. (**b**) Diffusion rates of SRB released from MSN-pDAEM2 (orange columns), MSN-pDAEM5 (green columns), and MSN-pDAEM15 (purple columns); B–G indicate the time intervals shown in (**a**). Time interval A corresponds to the desorption of SRB remaining at (or close to) the particle surface after the cleaning procedure.

To test the efficiency of the other controlling component of the system, the outer polymer shell, we tested nanoparticles with no pore modification but with three different values of polymer shell thickness. To follow the release of SRB from the MSN-pDAEM2 (2.1 wt% polymer/silica), MSN-pDAEM5 (5.1 wt% polymer/silica), and MSN-pDAEM15 (15 wt% polymer/silica), the SRB-loaded nanoparticles were dispersed in PBS solution at pH = 5 and placed the top compartment of the fluorescence cuvette, and the fluorescence intensity was monitored for 8.3 h (Figure 6). The addition of basic solution at specific time intervals increased the pH from 5 to 9 in the dialysis chamber, while in-between additions, the equilibrium is restored, and the pH in the device recovers to ca. pH 5 (Figure 6). The initial slope in each sample results from residual SRB adsorbed to the outer surface (Figure 6a, zone A) and was not considered when calculating the release rates (Figure 6b).

The conformational response of the pDAEM shell to pH changes results from the protonation/deprotonation of its tertiary amines (Figure 4). At acidic pH, the amines are protonated, and the polymer is positively charged in an expanded conformation. When the pH increases (after base addition), the amines deprotonate, and the polymer loses the charge and collapses into a globule-like conformation. Both the conformation of the polymer and its interaction with the cargo contribute to its release or retention. In the case of SRB (an anionic molecule), the interaction with the polymer shell is much stronger at low pH (cationic chains) due to electrostatic attraction, resulting in efficient retention of the cargo in the polymer network. Upon increasing the pH, the electrostatic interaction between the polymer and the cargo is lost, and the collapse of the polymer shell squeezes the cargo out. The results of the release experiments (Figure 6) clearly show the sponge-like squeezing behavior of the system for all the polymer shell thicknesses tested.

With the system at pH 5, the SRB diffuses from the MSN pores and is retained in the polymer shell due to the hindered diffusion in the expanded polymer network and the electrostatic interaction of the negatively charged cargo with the positively charged protonated polymer shell. Upon addition of base (Figure 6a, zones B, D, and F), the fluorescent intensity sharply increases due to release of the cargo by loss of the electrostatic interaction between the SRB and the protonated tertiary amines of the polymer shell, and also to the collapse of the polymer shell that squeezes the cargo out.

Following the fast cargo release period, mixing between the top (pH 9) and the (much larger) bottom (pH 5) compartments, decreases the pH from 9 back to ca. pH 5. This leads to the conformational transition of the collapsed non-charged shell to an expanded positively charged shell and to the SRB molecules diffusing from the MSN pores being retained at the polymer shell, stopping the release of the cargo (Figure 6a, zones C, E and G).

The release rates calculated for the different intervals (Figure 6b) show that the system behaves like an ON:OFF release system, with an efficiency that depends on the thickness of the polymer shell. To compare the performance of the different samples, we calculated the ratio of the release rates between the ON and OFF (basic and acid) states. The MSN-pDAEM15 sample presented the better performance (ON:OFF ratio of 13), with the other two samples showing lower ON:OFF ratios: 9 for MSN-pDAEM5 and 5 for MSN-pDAEM2 (this later ratio is similar to that obtained for experiments with fixed pH values, Figure S5). The obtained ratios compare very favorably with recently reported pH-controlled nanocarriers (Table S2, Supporting Information), although other systems do not follow the release in real-time and report only cumulative release values at different pH values.

Unlike most systems, our nanocarriers release the cargo at high pH and retain the cargo at low pH. Additionally, these nanocarriers have the advantage of operating in a pH range around the physiological pH (Table S2, Supporting Information).

The different release rate ratios obtained for these systems are explained by the thicker polymer shells providing a more efficient diffusion barrier and having more charged groups at acid pH (with the stronger interaction with the anionic cargo), resulting in lower leakage of the cargo in the OFF (acid pH) state. MSN-pDAEM5 shows larger release rates when the pH is increased than MSN-pDAEM2 (Figure 6b) because having a thicker shell retains more cargo at low pH. However, the same trend is not observed when the polymer shell is further increased to 15 wt% (MSN-pDAEM15 sample). Here, the increase in shell thickness does not translate into larger cargo release because the shell probably acts as an obstacle to the release of the cargo upon the collapse of the polymer, especially to the cargo further away from the shell surface. For this reason, MSN-pDAEM15 have lower release rates than MSN-pDAEM5 (Figure 6b).

Our experiments show that increasing the polymer shell thickness only increases the system ON:OFF performance to a point because the thicker polymer shell acts as a barrier to the release of the cargo upon the collapse of the polymer (evidenced by the results for MSN-pDAEM15). The solution is thus to couple the pH-responsive polymer shell to pore functionalization.

#### 3.2.3. Dual Release Control Mechanism: Functionalized Pores and Polymer Shell

By combining the two control release strategies, pH-responsive polymer shell and pore functionalization, with groups that have a strong interaction with the cargo, we reduce cargo leakage in the OFF state while maintaining the pumping-out release mechanism. This is important, for example, when off-target release is not acceptable in controlled drug delivery.

**Figure 7:**
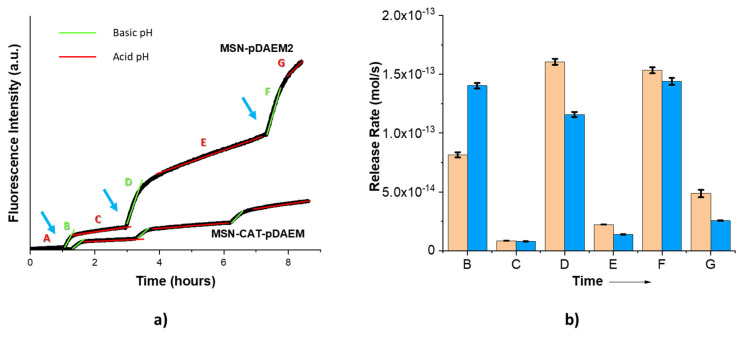
(**a**) Fluorescence intensity of SRB released from SRB-loaded MSNpDAEM2 and MSN-CAT-pDAEM (solid black lines), diffusing across the dialysis membrane (measured in the bottom compartment of the cuvette in PBS). The system is followed continuously for 8.3 h (λ_exc_ = 566 nm, λ_em_ = 589 nm). The blue arrows indicate additions of base solution in the top compartment of the cuvette, green lines (zones B, D, and F), and red lines (zones C, E, and G) indicate basic and acid pH periods, respectively. (**b**) Cargo release from MSN-pDAEM2 (orange columns) and MSN-CAT-pDAEM (blue columns); B–H indicate the time intervals shown in (**a**). Time interval A corresponds to the desorption of SRB remaining at (or close to) the particle surface after the cleaning procedure.

After pore functionalization with CAT, a relatively low thickness pDAEM shell was grown by RAFT to avoid the undesired decrease in release rate on the ON state. The obtained nanoparticles, MSN-CAT-pDAEM, have a polymer shell of 1.4 wt% (polymer:silica), comparable in thickness to MSN-pDAEM2. Release measurements performed as for the other hybrid systems (Figure 7a) show that at basic pH (ON state), the release rates are similar for the pore-functionalized and non-functionalized systems (Figure 7b), since both systems have similar polymer content in the shell. However, at acid pH (OFF state), MSN-CAT-pDAEM shows lower release rates than MSN-pDAEM2, indicating better cargo retention. In fact, the introduction of CAT almost doubles the retention efficiency of the system, with the ON:OFF release rate ratio increasing from 5 for MSN-pDAEM2 to 8 for MSN-CAT-pDAEM, an ON:OFF ratio similar to that obtained for MSN-pDAEM5 (bare pores).

This shows that internal pore functionalization in combination with a relatively thin stimuli-responsive polymer shell provides much better ON:OFF release performance on smart hybrid nanocarriers for pH actuated cargo release than using a thicker polymer shell alone.

## 4. Conclusions

Our novel nanocarrier for pH-actuated modulated controlled release, based on a hybrid mesoporous silica core with functionalized pore walls and a pH-responsive polymer shell (pKa ≈ 6.5), shows excellent ON:OFF cargo release efficiency. Our system features exceptional retention of cationic cargo at low pH and a sponge-like burst release at higher pH. Unlike most systems in the literature, our nanocarriers release the cargo at high pH and retain the cargo at low pH, operating in a range around physiological pH.

Release control results from the expanded-collapsed conformation transition of the pH-responsive polymer shell and the interaction of the cargo with the polymer shell and the pore walls. At pH < pKa ≈ 6.5 (OFF), the electrostatic interaction of the cargo with the functionalized pore walls and the protonated amine groups of the extended polymer shell retain the cargo, resulting in very low leakage of the cargo. At pH > pKa ≈ 6.5 (ON), the electrostatic interaction of the polymer with the cargo is lost (due to the deprotonation of the polymer amine groups), and the gel-like shell collapses, expelling the cargo by a sponge-like squeezing effect that results in a very high release rate.

The ON:OFF performance of the system increases with the thickness of the polymer shell. However, if the shell is too thick, it acts as a diffusion barrier to releasing the cargo in the collapsed OFF state, resulting in lower release rates. The best design strategy is thus to functionalize the pores of the MSN core with a group that interacts with the cargo molecules to retain the cargo in combination with a relatively thin pH-responsive polymer shell that produces very high release rates in the ON state. The results obtained with MSN pore functionalization in combination with a stimuli-responsive polymer shell are extremely promising for the design of high-performance ON:OFF smart hybrid nanocarriers with excellent cargo retention at low pH and sponge-like fast release at lower pH. This system can be easily tuned for other types of cargo and modified with groups for targeting tumors or other locations. Our novel nanocarrier design has huge potential for application in the controlled delivery of drugs and other bioactive cargo.

## Supplementary Materials

The following are available online at https://www.mdpi.com/article/10.3390/pharmaceutics13050716/s1, Figure S1, MSNs nitrogen sorption isotherm; Figure S2, determination of RAFT concentration at the MSNs surface by UV-Vis spectroscopy; Table S1, values of polymer incorporated per mass of nanoparticles in the different polymerization reactions; Figure S3, TEM image of the MSN-pDAEM15 showing the polymer shell; Figure S4, hydrodynamic diameter distribution of MSN-pDAEM at low pH, obtained by NTA; Figure S5, fluorescence intensity of SRB released from SRB-loaded MSN-pDAEM2 in PBS at pH 5 and pH 9; Table S2, comparison between our ON:OFF ratio results and data from other published systems.
