# Supplementary material for: Mesoporous Silica Nanoparticles Modified inside and out for ON:OFF pH-Modulated Cargo Release
Source: Pharmaceutics. 2021 May 13;13(5):716. doi: 10.3390/pharmaceutics13050716 (PMC8153141; doi:10.3390/pharmaceutics13050716)
Supplement: Supplementary file 1 [file pharmaceutics-13-00716-s001.zip › pharmaceutics-1148564-supplementary.pdf]

pharmaceutics

Supplementary Materials: Mesoporous Silica Nanoparticles Modified inside and out for ON:OFF pH-Modulated Cargo Release

José L. M. Gonçalves, Ana Beatriz C. Lopes, Carlos Baleizão * and José Paulo S. Farinha *

Contents

(A) MSNs Nitrogen Sorption Isotherm ... 2
(B) Determination of the amount of RAFT agent on the MSNs ... 2
(C) Polymer incorporation values ... 3
(D) TEM image of Hybrid MSN ... 3
(E) Size distribution data of NTA analysis ... 4
(F) Release from SRB-loaded MSN-pDAEM2 at pH 5 and pH 9 ... 5
(G) Comparison with nanocarriers reported in the literature ... 6
(H) REFERENCES ... 7

(A) MSNs Nitrogen Sorption Isotherm

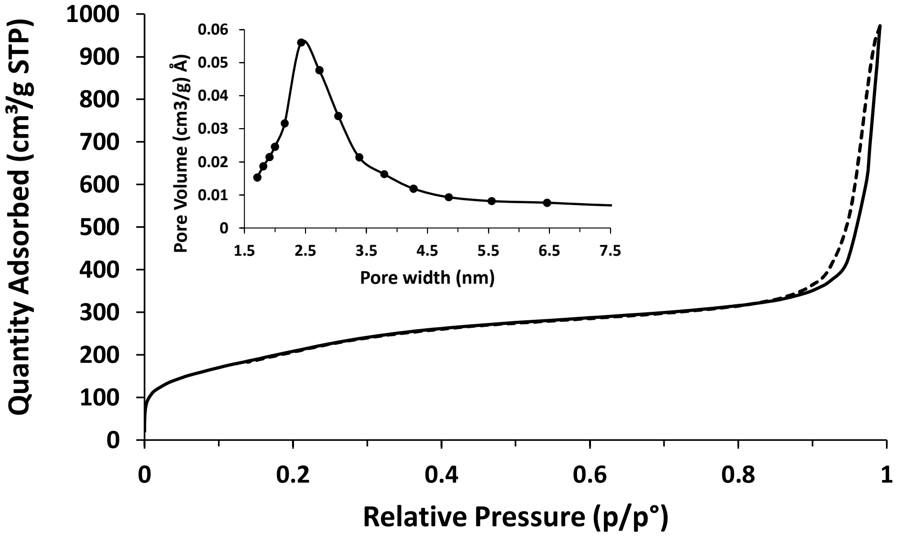

Figure S1. Nitrogen adsorption (solid line)-desorption (dotted line) isotherm for the MSNs, and corresponding pore size distribution (inset).

(B) Determination of the amount of RAFT agent on the MSNs

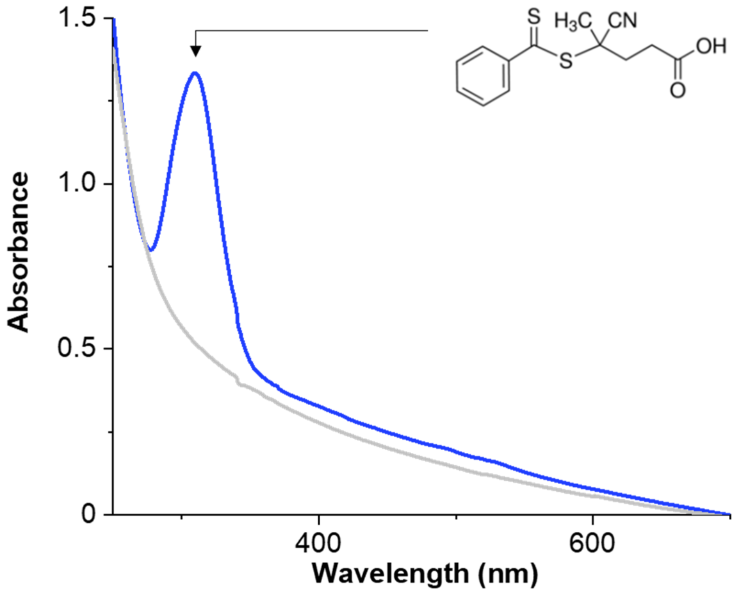

Figure S2. The amount of RAFT agent at the MSNs surface was calculated by subtracting the light scattering contribution (measured for bare MSNs, grey curve), from the absorption spectrum of MSN-CTA (blue curve) at the of absorption maximum wavelength (305nm).

(C) Polymer incorporation values

Table S1. Values of polymer incorporated per mass of nanoparticles in the different polymerization reactions.

[DAEM] (M) \ [CTA]/[AIBN]	5/1	10/1
**0.5**	0.9 wt%	2.1 wt%
**2**	1.9 wt%	5.1 wt%

(D) TEM image of Hybrid MSN

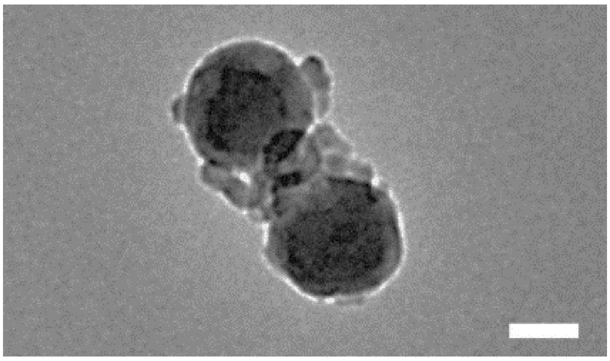

Figure S3. TEM image of the MSN-pDAEM15 showing the polymer shell. Scale bar = 50 nm.

(E) Size distribution data from NTA analysis

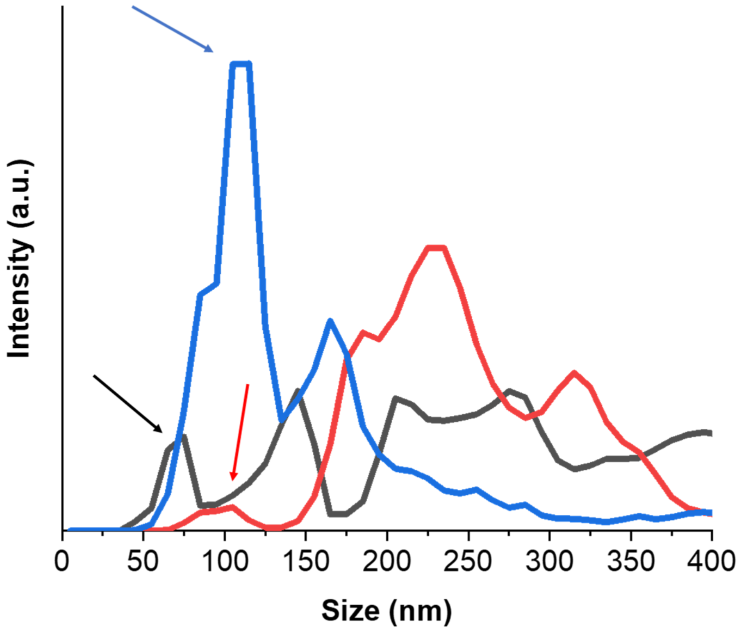

Figure S4. Hydrodynamic diameter distribution of MSN-pDAEM2 (black curve), MSN-pDAEM5 (red curve) and MSN-pDAEM15 (blue curve) at low pH, obtained by NTA. The lower value peak of each distribution corresponds to the diameter of the individual hybrid nanoparticles. The peaks at larger values are due to particle flocculation during the measurement.

(F) Release from SRB-loaded MSN-pDAEM2 at pH 5 and pH 9

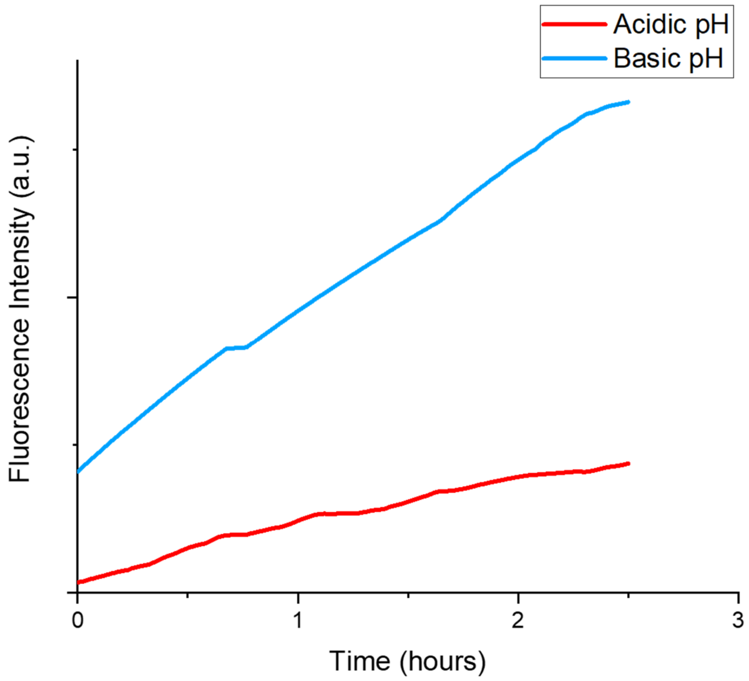

Figure S5. Fluorescence intensity of SRB released from SRB-loaded MSN-pDAEM2 in PBS at pH 5 (red line) and pH 9 (blue line). The released SRB diffuses across the dialysis membrane and is measured in the bottom compartment of the cuvette (λexc = 566 nm, λem = 589 nm). The release rate ratio is 4.5 and the release amount ratio is 4.0 after 2.5 hours.

(G) Comparison with nanocarriers reported in the literature

Table S2. Comparison of ON:OFF release ratios at 2h30 between our system MSN-pDAEM15 and recently reported controlled release nanocarriers using pH-responsive polymers.

System	Core diameter (nm)	pH (ON)	pH (OFF)	ON:OFF release ratio (at 2h30)	Ref
**Core-Shell**<br>Core: MSNs<br>Shell: pDAEM	45 ± 9	9	5	6	MSN-pDAEM15
**Core-Shell**<br>Core: MSNs-CAT<br>Shell: pDAEM	45 ± 9	9	5	5	MSN-CAT-pDAEM
**Core-shell**<br>Core: silica nanoparticles<br>Shell: poly(acrylic acid)	130	3	7.4	2	[1]
**Core-shell**<br>Core: MSNs<br>Shell: poly(vynil pyridine)	40	5.8	7.4	5	[2]
**Core-shell**<br>Core: MSNs<br>Shell: poly(*N*,*N*-dimethylaminoethyl methacrylate)	80 ± 16	2	7.4	3	[3]
**Core-shell**<br>Core: silica nanoparticles<br>Shell: poly(2-(diethylamino)ethyl methacrylate)	22-25	5.5	7.4	1	[4]
**Hybrid hollow nanogel**<br>Poly(*N*,*N*-dimethylaminoethyl methacrylate)	10-15	3.5	7.5	2	[5]

References

1. Wang, Q.; Jin, X.Q.; Sun, J.H.; Bai, S.Y.; Wu, X. PAA-grafted surface and fractal feature of dense nanosilica spheres for ibuprofen delivery. *Mater. Chem. Phys.* **2017**, *195*, 213–223.
2. Abbaszad Rafi, A.; Mahkam, M.; Davaran, S.; Hamishehkar, H. A Smart pH-responsive Nano-Carrier as a Drug Delivery System: A hybrid system comprised of mesoporous nanosilica MCM-41 (as a nano-container) & a pH-sensitive polymer (as smart reversible gatekeepers): Preparation, characterization and in vitro releas. *Eur. J. Pharm. Sci.* **2016**, *93*, 64–73.
3. Zhou, H.; Wang, X.; Tang, J.; Yang, Y.-W. Surface Immobilization of pH-Responsive Polymer Brushes on Mesoporous Silica Nanoparticles by Enzyme Mimetic Catalytic ATRP for Controlled Cargo Release. *Polymers.* **2016**, *8*, 277.
4. Xu, L.; Li, H.-L.; Wang, L.-P. PH-Sensitive, Polymer Functionalized, Nonporous Silica Nanoparticles for Quercetin Controlled Release. *Polymers.* **2019**, *11*, 2026.
5. Hajebi, S.; Abdollahi, A.; Roghani-Mamaqani, H.; Salami-Kalajahi, M. Hybrid and hollow Poly(*N*,*N*-dimethylaminoethyl methacrylate) nanogels as stimuli-responsive carriers for controlled release of doxorubicin. *Polymer.* **2019**, *180*, 121716.
